# Digital Patient–Reported Cancer Symptom Management: Comparison of Black and White Participants

**DOI:** 10.1002/cam4.71026

**Published:** 2025-07-09

**Authors:** Mary Gullatte, Bridget Nicholson, Elizabeth Sloss, Natalya Alekhina, Ann Marie Moraitis, Eli Iacob, Gary Donaldson, Kathi Mooney

**Affiliations:** ^1^ Emory University Atlanta Georgia USA; ^2^ University of Utah Salt Lake City Utah USA; ^3^ University of Rhode Island Providence Rhode Island USA

**Keywords:** electronic patient reporting, ePRO, oncology, remote patient monitoring, symptom science

## Abstract

**Introduction:**

Digital symptom monitoring effectively reduces symptom burden in cancer patients receiving treatment. However, concerns persist about whether digital interventions are equitable across racial groups, and limited studies have reported on differences between racial groups. This secondary analysis compared engagement, satisfaction, and cancer symptom reduction benefits in Black and White participants utilizing an electronic capture of patient‐reported outcome (ePRO) reporting system, symptom care at home (SCH), throughout chemotherapy.

**Methods:**

Participants undergoing cancer treatment at a comprehensive cancer center reported daily on the 11 common oncology symptoms via electronic patient reporting (ePRO) on a scale of 0–10. The mean symptom burden over time was compared between Black and White patients. End‐of‐study patient satisfaction ratings were completed and compared between Black and White patients receiving chemotherapy at a comprehensive cancer center in Atlanta, Georgia, USA.

**Results:**

Of the 357 self‐identified participants, 239 (66.95%) were Black, and 118 (33.05%) were White. Black participants were more likely to be female, have breast cancer, not currently married, and have less than a 4‐year college degree compared to Whites. Black participants had lower adherence to daily symptom reporting (65.25%; SD = 26.90 vs. 72.75%; SD 22.45; *p* = 0.01), but both groups reported the majority of days. The intervention produced a significant improvement in symptom burden (*p* < 0.001), with a mean improvement of 0.14 symptom burden points per week with equal benefit between Blacks and Whites (*p* = 0.27). Overall satisfaction was high for both groups, with a trend toward higher Black satisfaction (*p* = 0.06).

**Conclusion:**

Carefully designed digital health technologies can be acceptable and beneficial in reducing symptom burden for both Blacks and Whites. All patients, but particularly Black patients, may benefit from tailored encouragement strategies to report their symptoms. This paper provides evidence that ePRO reporting systems do not perpetuate inequity but, in fact, benefit cancer patients across racial groups.

## Graphical abstract

Carefully designed digital health technologies can be acceptable and beneficial in reducing symptom burden for both Blacks and Whites. All patients, but particularly Black patients, may benefit from tailored encouragement strategies to report their symptoms consistently.

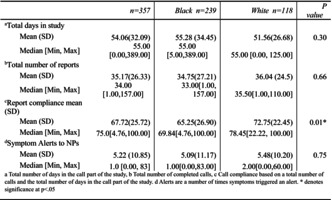

## 1 Introduction

Digital symptom management systems that utilize electronic capture of patient‐reported outcomes (ePROs) in individuals receiving cancer treatment reduce symptom burden and improve quality of life [1, 2, 3, 4]. These technologies have advanced the systematic monitoring and delivery of symptom care tailored to the individual's real‐time symptom experience between clinic visits. Due to their automation, ePRO digital health systems are also efficient and can provide automated self‐management feedback with escalation to clinician intervention when needed, thus making them highly scalable and bridging current supportive care access issues. Most studies of user acceptability report high satisfaction. However, concerns have been raised that this transformative technology has the potential to also create disparities among racial groups due to difficulty in owning, engaging with, or utilizing the technology, resulting in unequal benefits in achieving symptom reduction [4, 5, 6]. There is some evidence that disparities exist in the use of ePROs among Black patients, with lower engagement in initiating ePRO reporting [7, 8]. Other studies have demonstrated that ePRO reporting adherence was not different based on race [5, 6]. However, there may be nuanced differences between what Blacks and Whites report about using ePRO symptom systems [4]. It is important that digital technologies not add to the disparities in care that already exist. For example, there is evidence that Blacks experience higher symptom burden and greater symptom severity than their White counterparts [9, 10, 11]. Higher cancer symptom burden also translates into higher rates of hospital readmission from the emergency department for Blacks [12] as well as longer hospital stays than Whites [13]. While ePRO symptom monitoring and management systems show promise for lowering symptom burden and decreasing acute care utilization [5], success is contingent upon knowing whether ePRO systems are effective in patient engagement and lowering symptom burden across all populations. There are limited studies of ePRO digital health technology use and its value for Black patients with cancer in terms of satisfaction, engagement, and symptom burden reduction. This secondary analysis compared ePRO reporting engagement, satisfaction, and symptom reduction benefit in Black and White cancer patients utilizing data from a recent study of symptom care at home (SCH) [14], a multicomponent ePRO symptom monitoring and management system. This study adds an integral understanding of both ePRO use and benefit among Black and White participants.

## 2 Methods

### 2.1 Parent Study Design and Participants

In the parent RCT, the components of the SCH system were evaluated to determine the necessity of each of the individual intervention elements to achieve the improved symptom burden reduction found in the prior efficacy study [1]. The five‐group design included: (1) automated self‐management coaching, (2) automated self‐management coaching with an activity tracker, (3) nurse practitioner (NP) follow‐up when symptoms exceeded alert thresholds but no self‐management coaching, (4) NP follow‐up when symptoms exceeded alert thresholds utilizing the SCH decision support system but no self‐management coaching, and (5) all intervention components, or the complete SCH. The 757 participants were patients with cancer, 18 or older, and beginning a chemotherapy protocol either at the Huntsman Cancer Institute at the University of Utah in Salt Lake City, Utah, USA, or Winship Cancer Institute of Emory University, including patients from Grady Hospital in Atlanta, Georgia, USA. Patients were consented and enrolled after IRB approval was obtained at the two recruitment sites. Participants reported daily on the presence and severity of 11 common disease and treatment‐related symptoms using interactive voice response (IVR) telephone technology that transmits digital data through the telephone network and does not require internet or a smartphone [15]. Symptoms reported at moderate‐severe levels (4 or > on a 0–10 scale) received immediate automated, tailored self‐management coaching and/or nurse practitioner follow‐up, depending upon group assignment. Results from the RCT published elsewhere [14] showed maximal decrease in mean symptom burden over time in Group 5 that included the complete SCH intervention.

### 2.2 Setting

While the parent study had two sites, Utah and Georgia, this secondary analysis examined data only from the Georgia site due to the imbalance in races enrolled at the Utah site, with only one self‐identifying Black participant and 370 self‐identifying White participants. In addition, we wanted to decrease potential confounders because each site had separate study teams, including state‐based nurse practitioners that interacted with participants based on different state regulations for prescribing medication in response to symptom alerts.

### 2.3 Measures

#### 2.3.1 Demographic and Disease Characteristics

The parent study collected baseline participant‐reported demographic characteristics and electronic health record (EHR) abstracted disease‐related characteristics. Participants self‐identified their race as part of the baseline demographic questionnaire. Those identifying as either Black or White from the Georgia site were included in this secondary analysis.

#### 2.3.2 Symptom Reporting

Participants were asked to report symptom presence and severity (0–10 scale) daily, ideally before noon, so the nurse practitioner could follow‐up if symptoms warranted in the NP groups. Participants were encouraged to report 7 days per week, including holidays, for the full duration of their chemotherapy protocol or 6 months, whichever came first. If the participant was assigned to an NP care group, the follow‐up, if warranted, was also 7 days a week, including holidays. Single‐item measures were utilized to report symptom severity based on the prior efficacy study, the necessity to keep the reporting burden low while monitoring 11 symptoms daily, and because single measures are reliable and valid [16, 17].

#### 2.3.3 Symptom Burden

Symptom burden was derived from the daily participant symptom reports received in the SCH system.

#### 2.3.4 Satisfaction With SCH and Symptom Care

Participants completed a 16‐item yes/no and Likert scale survey about their experience and satisfaction in using the SCH system and the symptom care they received. Qualitative comments were also solicited. The survey, which could be completed through a REDCap survey or in person, was administered at the end of the study or when a participant withdrew.

### 2.4 Statistical and Qualitative Analysis

We compared study participants who self‐identified as Black or White to determine if symptom experience, satisfaction, and engagement with the SCH platform and the degree of symptom burden reduction over time were similar across the two groups. Descriptive statistics were used to compare demographic and disease‐related variables. Student *t*‐tests and chi‐square tests were utilized as appropriate. Symptom prevalence was determined by the number of reports received with 1 or more symptoms rated at a severity level of 4 or greater on the 0–10 scale. Reporting adherence was based on the proportion of participant reports received compared to expected. The mean summed daily symptom severity item scores across 11 symptoms comprised the overall symptom burden score. To evaluate the intervention's effectiveness at reducing symptom burden, we regressed the symptom burden score on the study day, incorporating a statistical term that realistically controls for persistent individual differences and analyzed by the categorical variable of self‐identified race. Under this simple model, a negative regression coefficient indicates improvement (reduced burden over time), while a positive coefficient denotes worsening (higher burden over time). Participant experience and satisfaction with SCH and symptom care were analyzed via descriptive analysis, independent *t*‐test, independent Mann–Whitney *U* tests, and chi‐square.

## 3 Results

### 3.1 Demographic and Disease Characteristics

In the parent study, 467 participants were enrolled at the Georgia site, and 370 made one or more symptom reports to the SCH system. Thirteen participants (3.51%) either did not identify a race or identified as a race other than Black or White. Of the 357 self‐identified Black or White participants, 239 (66.95%) were Black, and 118 (33.05%) were White. Table 1 compares the demographics of the Black and White participants. Black participants were more likely to be female, not currently married, have less than a 4‐year college degree, and have a diagnosis of breast cancer compared to White participants. Black and White groups were not different in the stage of their cancer, with a majority having stages III or IV disease. The mean age was approximately 59 years, which did not differ between groups. There were no differences in the distribution of participants in the parent study intervention groups by race.

**TABLE 1:** Demographic characteristics of patients who reported symptoms through the SCH system by race.

	Categories	Total (*n* = 357)	Black (*n* = 239)	White (*n* = 118)	*p*
Age	Mean (SD)	59.30 (12.14)	59.14 (11.51)	59.63 (13.8)	0.07
Gender	Male (%)	128 (35.85%)	68 (28.45%)	60 (50.85%)	< 0.001
	Female	229 (64.15%)	171 (71.55%)	58 (49.15%)	
Hispanic	No	349 (97.76%)	234 (97.91%)	115 (97.46%)	0.95
	Yes	5 (1.41%)	3 (1.26%)	2 (1.69%)	
	Missing	3 (0.84%)	2 (0.84%)	1 (0.85%)	
Marital status	Divorced/separated	73 (20.45%)	61 (25.52%)	12 (10.17%)	< 0.001
	Married/living with a partner	173 (48.46%)	87 (36.40%)	86 (72.88%)	
	Never married/single	92 (25.77%)	76 (31.80%)	16 (13.56%)	
	Widowed	17 (4.76%)	13 (5.44%)	4 (3.39%)	
	Missing	2 (0.56%)	2 (0.84%)	0 (0.0%)	
Education	Less than high school diploma	24 (6.72%)	19 (7.95%)	5 (4.24%)	0.03
	Bachelor	96 (26.85%)	55 (23.01%)	41 (34.7%)	
	Graduate degree	41 (11.48%)	22 (9.21%)	19 (16.10%)	
	High school or equivalent	91 (25.49%)	66 (27.62%)	25 (21.18%)	
	Some college/associate degree	103 (28.85%)	75 (31.30%)	28 (23.73%)	
	Missing	2 (0.56%)	2 (0.84%)	0 (0.00%)	
Income	$< 25,000	61 (17.09%)	47 (19.7%)	14 (11.86)	< 0.001
	$25,000–$49,999	140 (39.22%)	90 (37.66%)	50 (42.37%)	
	$50,000–$74,999	65 (18.21%)	38 (15.90%)	27 (22.88%)	
	$75,000–$99,999	14 (3.92%)	4 (1.67%)	10 (8.47%)	
	$100,000–$124,000	3 (0.84%)	0 (0.00%)	3 (2.54%)	
	≥ $125,000	3 (0.84%)	1 (0.42%)	2 (1.69%)	
	Don't know/prefer not to answer	55 (15.41%)	49 (20.50%)	6 (5.08%)	
	Missing	16 (4.48%)	10 (4.18%)	6 (5.08%)	
Diagnosis	Breast	100 (28.01%)	85 (35.56%)	15 (12.71)	< 0.001
	Lung	50 (14.01%)	31 (12.97%)	19 (16.10%)	
	Colorectal	30 (8.40%)	25 (10.46%)	5 (4.24%)	
	Ovarian	21 (5.88%)	13 (5.44%)	8 (6.78%)	
	Pancreatic	21 (5.88%)	13 (5.44%)	8 (6.78%)	
	Other	135 (37.82%)	72 (30.13%)	63 (53.39%)	
Cancer stage	I	42 (11.76%)	31 (12.97%)	11 (9.32%)	0.06
	II	51 (14.29%)	37 (15.48%)	14 (11.86%)	
	III	83 (22.25%)	59 (24.69%)	24 (20.34%)	
	IV	154 (43.14%)	100 (41.84%)	54 (45.76%)	
	Unknown	25 (7.56%)	12 (5.02%)	15 (12.71%)	

^a^ Unless otherwise specified % follows *n*.

### 3.2 Engagement and Reporting Adherence

Of the total sample of patients enrolled (*n* = 467), 357 (76.45%) called into the system at least once. There was no significant difference between Blacks and Whites on participation by calling at least once (*n* = 239 out of 307, 77.85% versus *n* = 118 out of 141, 83.69%, *p* = 0.19).

Black participants had lower adherence to daily symptom reporting compared with Whites (65.25% (SD = 26.9) vs. 72.75% (SD 22.4); *p* = 0.01), but both groups reported the majority of days, and the difference was only 7.50%. There were no differences in average days on study for Blacks at 55.28 days (SD 34.4) and 51.56 days (SD 26.7) for Whites (*p* = 0.30).

### 3.3 Symptom Burden and Symptom Benefit From SCH

Overall, of the 357 patients who reported symptoms at least once, 269 (75.35%) endorsed one or more symptoms in the moderate‐severe range with no difference between the two racial groups overall and for any of the 11 individual symptoms tracked (*p* = 0.59) (see Table 2). There was no difference in the number of alerting symptom notifications sent to NPs for follow‐up, with an average of 5.09 (11.17) versus 5.48 (10.20) (*p* = 0.75) days of alerting for Blacks and Whites, respectively (see Table 3).

**TABLE 2:** Participants with at least one moderate or severe symptoms and reporting by race.

Symptom	Total		Black		White		*p*
	*n* = 357	%	*n* = 239	%	*n* = 118	%	
Any symptom	269	75.35	178	74.48	91	77.11	0.59
Pain	208	58.26	139	58.16	69	58.47	0.96
Fatigue	196	54.90	124	51.88	72	61.07	0.10
Trouble sleeping	169	47.34	108	45.18	61	51.69	0.25
Nausea/vomiting	134	37.54	90	37.66	44	37.29	0.95
Numbness/tingling	96	26.89	70	29.29	26	22.03	0.15
Sore mouth	88	24.65	53	22.18	35	29.66	0.12
Trouble thinking/concentrating	89	24.93	53	22.18	36	30.50	0.09
Diarrhea	76	21.28	49	20.50	27	22.88	0.61
Feeling nervous/anxious	64	17.93	37	15.48	27	22.88	0.09
Trouble breathing	63	17.65	40	16.74	23	19.49	0.52
Depressed mood	59	16.53	35	14.64	24	20.34	0.17

**TABLE 3:** Study days, reports and report compliance, symptom alerts.

	*n* = 357	Black *n* = 239	White *n* = 118	*p*
Total days in study^a^				
Mean (SD)	54.06 (32.09)	55.28 (34.45)	51.56 (26.68)	0.30
Median [min, max]	55.00 [0.00, 389.00]	55.00 [5.00, 389.00]	55.00 [0.00, 125.00]	
Total number of reports^b^				
Mean (SD)	35.17 (26.33)	34.75 (27.21)	36.04 (24.5)	0.66
Median [min, max]	34.00 [1.00, 157.00]	33.00 [1.00, 157.00]	35.50 [1.00, 110.00]	
Report compliance mean (SD)^c^				
Mean (SD)	67.72 (25.72)	65.25 (26.90)	72.75 (22.45)	0.01*
Median [min, max]	75.0 [4.76, 100.00]	69.84 [4.76, 100.00]	78.45 [22.22, 100.00]	
Symptom alerts to NPs^d^				
Mean (SD)	5.22 (10.85)	5.09 (11.17)	5.48 (10.20)	0.75
Median [min, max]	1.0 [0.00, 83]	1.00 [0.00, 83.00]	2.00 [0.00, 60.00]	

^a^ Total number of days in the call part of the study.

^b^ Total number of completed calls.

^c^ Call compliance based on a total number of calls and the total number of days in the call part of the study.

^d^ Alerts are a number of times symptoms triggered an alert.

* Denotes significance at *p* < 0.05.

We modeled symptom burden over time in days. Because of the five‐group design, we chose to model the group that received the full SCH intervention (automated self‐management coaching and NP follow‐up) because that was the intervention that proved most efficacious in the parent study and has been adopted for implementation going forward [14]. This group consisted of Blacks (*n* = 59) and Whites (*n* = 26). The rates of change for Blacks and Whites did not differ significantly (*p* = 0.27), so the time‐by‐race interaction term was eliminated from the model. Both races showed a highly significant (*p* < 0.001) mean improvement of 0.14 symptom burden points per week (or 0.02 points per day). The intervention produced a similar average benefit for Blacks and Whites.

### 3.4 Satisfaction With SCH

Upon study completion, participants were asked to rate their overall satisfaction with reporting, ease of using the SCH system, and their symptom care. Of all Black and White participants enrolled (*n* = 357), a total of 216 participants (60.50%) completed the end‐of‐study interview, specifically 143 Blacks (59.8%) and 73 (61.9%) Whites. When asked to rate overall satisfaction with SCH on a scale from 1 to 10 (1 = not at all satisfied and 10 = very satisfied), Black participants had a mean satisfaction score of 8.98 (SD 1.66) compared to Whites 8.50 (SD 1.90), with no difference between groups (*p* = 0.06) although a trend toward higher satisfaction for Blacks. Open‐ended satisfaction comments were consistent across Blacks and Whites and included positive interactions with the study staff, appreciation for the reliability and consistency of the nurse practitioner calls, the study's usefulness in managing symptoms, and a heightened sense of being cared for. For example, one participant stated, “I thought it was a really good system and it really helped me with my symptoms and offered good information on management.” Similarly, another participant stated, “… the nurses have been really sweet and helpful.” Improvement feedback included frustration with repetitive questions, a desire for fewer calls or more flexible timing, and the ability to fully express symptoms or customize responses (e.g., “There are some redundancies with the questions asked each day”). Several participants noted a web‐based option may be preferred: “I like the phone, but web‐based would not be bad,” but multiple participants noted the ease of the phone system. The main technology complaint was the inability to correct the system if a wrong answer was submitted, “Let the patient go back and correct an answer if you push the wrong answer.” Some participants mentioned poor health, forgetfulness, or technical challenges as obstacles that hindered their full participation.

Both groups found the SCH system easy to use, with 95.11% of Black and 93.06% of White participants responding, “quite a bit” or “very much so.” Additionally, 99.30% of Black and 91.78% of White participants found the instructions on how to use the system helpful. Participants found the questions both easy to understand and answer, with approximately 85%–93% of both groups reporting no difficulties.

When asked about the call frequency, approximately 36%–40% of both groups felt the calls were too frequent. However, 89%–90% of respondents were satisfied with the length of the calls. Additionally, 72.34% of Black and 72.98% of White participants liked the SCH system “quite a bit” or “very much so.” Regarding the likelihood of using SCH again, over 70% of Black and White participants agreed they would use the system again “quite a bit” or “very much so.” Similarly, using SCH to track symptoms was highly rated, with 69.93% of Black and 61.11% of White participants finding it “quite a bit” or “very much” helpful. In terms of participating in their care, both groups (77.46% of Black and 69.45% of White) felt SCH helped them “quite a bit” or “very much.” SCH was also seen as helpful in reminding participants to call their doctor or nurse when needed, with 76.22% of Black and 62.50% of White participants responding, “quite a bit” or “very much so.” For example, a Black participant elaborated, “The very few times I talked with the nurse from the study, she had very good information for me, and I was able to discuss with my doctor.” The majority of participants felt SCH helped them know what symptoms to discuss with their providers (68.53% of Black and 53.42% of White participants). Anecdotally, two participants, one Black and one White, shared that they occasionally took notes after SCH phone calls about topics to discuss with their providers.

Overall, responses were similar between Black and White participants, but a few statistically significant differences were observed. This included instructions on using the SCH system, keeping track of symptoms, reminders to call their oncology team, and knowing what symptoms to discuss with the team. In each case, Black participants provided more positive ratings, although the majority of responses were positive across groups (see Table 4). Open‐ended comments by participants were not different between the racial groups.

**TABLE 4:** Participant responses to end of study interview grouped by race.

**Q1: How satisfied were you with the phone calls? A ‘10’ means you were very satisfied. A ‘1’ means you were not satisfied at all *t* = (df 213) = (1.90), *p* = 0.06**		
**Response**	**Black (*n* = 143)**	**White (*n* = 72)**
Mean (SD)	8.98 (1.66)	8.50 (1.90)
**Q2: How easy did you find the SCH system to use? (*p* = 0.71)**		
**Response**	**Black (*n* = 143)**	**White (*n* = 72)**
Not at all	3 (2.09%)	1 (1.39%)
Somewhat	4 (2.80%)	4 (5.55%)
Quite a bit	20 (13.99%)	10 (13.89%)
Very much so	116 (81.12%)	57 (79.17%)
**Q3: Did you find the instructions on how to use the SCH system helpful? (*p* = 0.003)**		
**Response**	**Black (*n* = 143)**	**White (*n* = 73)**
No	1 (0.70%)	6 (8.22%)
Yes	142 (99.30%)	67 (91.78%)
**Q4: Did calling every day seem too often for you? *p* = 0.63**		
**Response**	**Black (*n* = 143)**	**White (*n* = 73)**
No	91 (63.64%)	44 (60.27%)
Yes	52 (36.36%)	29 (39.73%)
**Q5: Was the length of the call acceptable to you? *p* = 0.82**		
**Response**	**Black (*n* = 142)**	**White (*n* = 73)**
No	15 (10.49%)	7 (9.59%)
Yes	127 (88.81%)	66 (90.41%)
**Q6: How much did you like using the SCH phone system? (*p* = 0.35)**		
**Response**	**Black (*n* = 141)**	**White (*n* = 73)**
Not at all	2 (1.42%)	4 (5.48%)
Somewhat	36 (25.53%)	15 (20.55%)
Quite a bit	36 (25.53%)	26 (35.61%)
Very much so	67 (47.52%)	28 (38.36%)
**Q7: How likely is it that you would use the SCH phone system again if you had the chance? (*p* = 0.84)**		
**Response**	**Black (*n* = 143)**	**White (*n* = 73)**
Not at all	11 (7.69%)	7 (9.59%)
Somewhat	29 (20.28%)	11 (15.07%)
Quite a bit	26 (18.18%)	18 (24.66%)
Very much so	77 (53.85%)	37 (50.68%)
**Q8: How much did using SCH help you keep track of your symptoms? (*p* = 0.047)**		
**Response**	**Black (*n* = 143)**	**White (*n* = 73)**
Not at all	13 (9.09%)	11 (15.28%)
Somewhat	30 (20.98%)	18 (25.00%)
Quite a bit	27 (18.88%)	17 (23.61%)
Very much so	73 (51.05%)	27 (37.50%)
**Q9: How much did SCH help you feel like you were participating in your care? (*p* = 0.27)**		
**Response**	**Black (*n* = 142)**	**White (*n* = 72)**
Not at all	4 (2.82%)	7 (9.72%)
Somewhat	28 (19.72%)	15 (20.83%)
Quite a bit	39 (27.46%)	17 (23.61%)
Very much so	71 (50.00%)	33 (45.84%)
**Q10: How much help was SCH in reminding you to call your doctor or nurse if you had concerns? (*p* = 0.02)**		
**Response**	**Black (*n* = 143)**	**White (*n* = 72)**
Not at all	11 (7.69%)	21 (29.17%)
Somewhat	23 (16.09%)	6 (8.33%)
Quite a bit	39 (27.27%)	16 (22.22%)
Very much so29.2+	70 (48.95%)	29 (40.28%)
**Q11: Did SCH help you know what symptoms you might talk about with your doctor or nurse at your next clinic visit? (*p* = 0.01)**		
**Response**	**Black (*n* = 143)**	**White (*n* = 73)**
Not at all	17 (11.89%)	23 (31.51%)
Somewhat	28 (19.58%)	11 (15.07%)
Quite a bit	33 (23.08%)	15 (20.54%)
Very much so	65 (45.45%)	24 (32.88%)
**Q12: In general, how easy is it to reach your doctor or nurse directly to talk over symptom concerns? (*p* = 0.59)**		
**Response**	**Black (*n* = 138)**	**White (*n* = 73)**
Not easy	17 (12.32%)	6 (8.22%)
Somewhat easy	26 (18.84%)	11 (15.07%)
Quite easy	25 (18.12%)	19 (26.03%)
Very easy	70 (50.72%)	37 (50.68%)
**Q13: Do you think the SCH voice sounded more like a person or a computer? (*p* = 0.42)**		
**Response**	**Black or African American (*n* = 143)**	**White (*n* = 73)**
Person	78 (54.55%)	44 (60.27%)
Computer	65 (45.45%)	29 (39.73%)
**Q14: How much did the SCH conversation sound like a conversation you would have with your doctor or nurse? (*p* = 0.06)**		
**Response**	**Black (*n* = 141)**	**White (*n* = 73)**
Not at all	20 (14.18%)	16 (21.92%)
Somewhat	51 (36.17%)	27 (36.98%)
Quite a bit	34 (24.12%)	20 (27.40%)
Very much so	36 (25.54%)	10 (13.70%)
**Q15: Did you have a hard time understanding any of the questions? (*p* = 0.47)**		
**Response**	**Black (*n* = 143)**	**White (*n* = 73)**
Yes	14 (9.79%)	5 (6.85%)
No	129 (90.21%)	68 (93.15%)
**Q16: Did you have a hard time knowing how to answer any of the questions? (*p* = 0.51)**		
**Response**	**Black (*n* = 141)**	**White (*n* = 72)**
Yes	17 (12.06%)	11 (15.28%)
No	124 (87.94%)	61 (84.72%)

## 4 Discussion

While concern has been raised that digital health tools may perpetuate care inequities [18, 19], we found equivalent benefit and satisfaction with SCH for Blacks and Whites. This is consistent with a sensitivity analysis that we conducted on our original SCH efficacy study, although with that study, only 12.00% of the sample identified as Black [6, 15]. Both studies used IVR telephone–based technology in the SCH platform, overcoming the need for internet or smart phone skills and therefore may be easier to utilize than web‐ or app‐based approaches. Other platforms allowing both internet and phone‐based reporting found that reporting mode preferences vary, but participants in our study reported high satisfaction with the phone system [5]. We now offer patients app, web, and IVR telephone reporting to SCH, allowing patients to choose their preferred reporting mode. This is an important consideration in deploying digital health platforms.

Black participants had lower daily reporting rates than Whites, but still reported 65.25% of expected days and a rate only 7.50% lower than Whites. The reporting rate was sufficient to obtain equivalent symptom reduction benefit. We encourage daily reporting so that symptom fluctuations are identified early and managed before escalation that results in unnecessary suffering and urgent care or emergency department visits. Recent studies have found that daily reporting is optimal for detecting clinically significant symptom fluctuations [20, 21]. Limited studies have evaluated engagement and sustainability of ePRO symptom reporting. Further study is warranted to determine optimal strategies for encouraging initial and ongoing use of ePRO reporting, including considering approaches that address diverse populations' needs and preferences.

A key finding was that the complete SCH intervention was equally beneficial in reducing symptom burden for both Blacks and Whites. Moderate‐severe symptoms were common in both groups. Other research has found a higher cancer symptom burden in Blacks [9, 10, 11]; however, we found no difference in overall or individual symptom severity, perhaps due to intervention efficacy in systematically identifying and intervening for symptoms at moderate levels, successfully reducing symptom severity escalation. We did not find that the number of symptom reports requiring alerts to the NPs differed between the two groups. For equitable care, it is important that symptom monitoring captures the patient's experience and interventions to manage symptom escalations are effective across populations. Systematic ePRO implementation paired with effective self‐management coaching and clinician follow‐up for poorly controlled symptoms offers an efficient approach to overcoming current limitations to symptom care centered on clinic visits, patient calls to the oncology team, or urgent visits to address symptom escalations.

Both Blacks and Whites were highly satisfied with SCH and symptom care. While others have found racial differences in user experience and valuing of symptom ePRO reporting [4], we did not. There were some differences between Blacks and Whites about the usefulness of our instructions, tracking symptoms, or if SCH helped them to think about symptoms to talk about at a clinic visit; but, in each case (Table 4) the differences resulted in Blacks being more favorable than Whites. This suggests the digital symptom monitoring intervention yields high satisfaction in Whites but may offer added value and satisfaction as a symptom care approach for Blacks over the current clinic visits and patient‐initiated telephone triage approach.

This secondary analysis was limited by the design of the parent study, as originally, we did not propose an aim to compare Black and White outcomes and satisfaction specifically, and the study may be underpowered to detect this particular difference. Due to racial differences in enrollment, we were limited to a single site where we enrolled a significant number of Black and White participants. Since, to our knowledge, this is the largest comparison of Black and White participants utilizing cancer ePRO symptom reporting that analyzed symptom experience, outcomes, engagement, and satisfaction with ePRO digital technology, we felt it important to compare both satisfaction and engagement. Our findings demonstrated equitable benefit and satisfaction based on race, but further study should consider both larger sample sizes and the wider context of social determinants of health for which race may be a proxy.

In summary, this analysis offers evidence that the complete SCH intervention was equally beneficial in reducing symptom burden for both Blacks and Whites. The combination of ePRO symptom monitoring systems with a systematic response to elevated symptom severity equitably benefits both Black and White patients receiving cancer therapies. Further research on racial preferences in both initial and long‐term engagement approaches would benefit patients engaged in digital health ePRO reporting. Both Black and White patients expressed high satisfaction and saw value in digital symptom monitoring and care. Using these systems may contribute to reducing racial disparities in cancer symptom care.

## Author Contributions

**Mary Gullatte:** conceptualization (equal), data curation (equal), formal analysis (equal), investigation (equal), methodology (equal), project administration (equal), writing – review and editing (equal). **Bridget Nicholson:** conceptualization (equal), data curation (equal), writing – original draft (equal), writing – review and editing (equal). **Elizabeth Sloss:** validation (equal), visualization (equal), writing – original draft (equal), writing – review and editing (equal). **Natalya Alekhina:** validation (equal), visualization (equal), writing – original draft (equal), writing – review and editing (equal). **Ann Marie Moraitis:** validation (equal), visualization (equal), writing – original draft (equal), writing – review and editing (equal). **Eli Iacob:** data curation (equal), formal analysis (equal), methodology (equal), resources (equal), validation (equal). **Gary Donaldson:** data curation (equal), formal analysis (equal), methodology (equal), validation (equal). **Kathi Mooney:** conceptualization (equal), data curation (equal), formal analysis (equal), funding acquisition (equal), investigation (equal), methodology (equal), resources (equal), supervision (equal), validation (equal), visualization (equal), writing – original draft (equal), writing – review and editing (equal).

## Ethics Statement

The funder had no role in the design and conduct of the study; collection, management, analysis, and interpretation of the data; preparation, review, or approval of the manuscript; and decision to submit the manuscript for publication. Informed consent was obtained from all individual participants included in the study in accordance with the Declaration of Helsinki. This study was approved by the Institutional Review Boards at the Huntsman Cancer Institute at the University of Utah in Salt Lake City, Utah, USA, and the Winship Cancer Institute of Emory University.

## Conflicts of Interest

Drs. Mooney and Nicholson have previously consulted for Reimagine Care ending in 2023. Dr. Nicholson consults for Daymark Oncology. All other authors have nothing to disclose.

## Supporting information


Appendix S1

## Data Availability

Data will be available from the corresponding author upon request, along with a study proposal and completion of a satisfactory data management plan.
